# High Resolution Measurement of Light in Terrestrial Ecosystems Using Photodegrading Dyes

**DOI:** 10.1371/journal.pone.0075715

**Published:** 2013-09-17

**Authors:** Javier Roales, Jorge Durán, Heather A. Bechtold, Peter M. Groffman, Emma J. Rosi-Marshall

**Affiliations:** 1 Departamento de Sistemas Físicos, Químicos y Naturales, Universidad Pablo de Olavide, Sevilla, Spain; 2 Cary Institute of Ecosystem Studies, Millbrook, New York, United States of America; University of Saskatchewan, Canada

## Abstract

Incoming solar radiation is the main determinant of terrestrial ecosystem processes, such as primary production, litter decomposition, or soil mineralization rates. Light in terrestrial ecosystems is spatially and temporally heterogeneous due to the interaction among sunlight angle, cloud cover and tree-canopy structure. To integrate this variability and to know light distribution over time and space, a high number of measurements are needed, but tools to do this are usually expensive and limited. An easy-to-use and inexpensive method that can be used to measure light over time and space is needed. We used two photodegrading fluorescent organic dyes, rhodamine WT (RWT) and fluorescein, for the quantification of light. We measured dye photodegradation as the decrease in fluorescence across an irradiance gradient from full sunlight to deep shade. Then, we correlated it to accumulated light measured with PAR quantum sensors and obtained a model for this behavior. Rhodamine WT and fluorescein photodegradation followed an exponential decay curve with respect to accumulated light. Rhodamine WT degraded slower than fluorescein and remained unaltered after exposure to temperature changes. Under controlled conditions, fluorescence of both dyes decreased when temperatures increased, but returned to its initial values after cooling to the pre-heating temperature, indicating no degradation. RWT and fluorescein can be used to measure light under a varying range of light conditions in terrestrial ecosystems. This method is particularly useful to integrate solar radiation over time and to measure light simultaneously at different locations, and might be a better alternative to the expensive and time consuming traditional light measurement methods. The accuracy, low price and ease of this method make it a powerful tool for intensive sampling of large areas and for developing high resolution maps of light in an ecosystem.

## Introduction

Solar radiation is a key factor associated with terrestrial ecosystem processes. For instance, the amount of exposure to sunlight can determine rates of primary production by photosynthesis [1,2] and litter decomposition due to photodegradation [3–5], which then influences soil mineralization rates [6,7]. Light in terrestrial ecosystems is spatially and temporally heterogeneous [8,9]. Tree-canopy traits, such as height, leaf shape or density determine light regimes, and there are obvious differences in incoming light under closed canopies, open canopies such as grassland ecosystems, or canopy gaps where light is patchy. Light heterogeneity may be especially significant in canopies that contain gaps or other features such as sunflecks, resulting in patchy light patterns that are of great ecological importance given that available light at small scales can significantly affect whole-system photosynthesis or leaf decay [10]. Changes in the angle of sunlight, which occur daily and annually, and cloud cover, can also interact with tree-canopy structure further increasing variability [9,11,12].

While in some cases single measurements of light in grasslands and beneath closed canopies give an acceptable approach to the nature of light regimes, measuring temporally or spatially heterogeneous light would better capture the light that is available for photosynthesis [12,13]. However, to do this, a high number of measurements are needed to integrate temporal and spatial variability and to obtain an accurate assessment of light distribution over time and space. Tools to measure light, such as light meters or hemispherical photographs, are limited, usually expensive, and allow only one or a small number of measurements at a time [14]. In particular, measuring and mapping heterogeneous light using light meters involves the simultaneous use of numerous devices (i.e. light meters and data loggers). The alternative analysis of hemispherical photographs has the added disadvantage of being a time consuming and sometimes inaccurate process [15]. Given that a single light measurement may be influenced by solar position, vegetation, and cloud cover, measuring ecologically relevant patterns of light for short-term studies requires many measurements and may result in a lot of time of data processing [15]. Thus, an inexpensive and easy-to-use tool that is sensitive to photosynthetically active radiation (PAR) is needed.

Recently, Bechtold et al. [16] developed a new method for measuring light in aquatic ecosystems using photodegrading fluorescent organic dyes, which are commonly used as tracers in stream ecosystem research [17,18]. These dyes feature a high fluorescence quantum yield that facilitates light estimation by determining rate of photo-degradation when exposed to light [19–21]. Results from the new method were highly correlated with estimates of accumulated light produced by commercially available PAR sensors. These dyes are cheap and easy to use, allowing extensive and simultaneous measurements of light over relatively large areas and integrate variables that influence the quantity and quality of incoming light. The new method was found to be useful to obtain integrated estimates of incoming solar radiation in streams [16,22], but it has not been tested in terrestrial ecosystems. Measuring the natural variability of light in terrestrial systems should be sensitive enough to capture rapid changes in light in multiple situations, e.g., from full sunshine to shade [23,24]. Some organic dyes can degrade or modify their fluorescence properties under high or low temperatures [25–27]. Although the method presented in Bechtold et al. [16] explored the influence of temperature on degradation in aquatic applications, terrestrial ecosystems often have more extreme and variable temperatures than aquatic systems, making further testing necessary.

There have been other attempts in the past to use inexpensive products, such as diazonium salts on ozalid paper [28,29] or photochemical methods [30,31] to monitor solar energy in terrestrial systems. Although the degradation of these other products has been demonstrated to be related to light exposure, the superiority of the method proposed in this study is based on the fact that photodegrading dyes are non-toxic, waterproof, inexpensive, easy to find and measure, but most importantly, they can integrate sunlight over longer (weeks) and more variable (from minutes to hours) time frames compared to these other methods (minutes to hours).

Our objective was to quantify the effectiveness of photodegrading dyes for measuring accumulated light across varying light and temperature conditions in terrestrial ecosystems. We modified methods from Bechtold et al. [16] using the same two photodegrading organic dyes, rhodamine WT (RWT) and fluorescein. We hypothesized that in terrestrial systems: (i) decay of RWT and fluorescein dyes exposed to natural sunlight will be correlated with accumulated light measured with PAR quantum sensors, and (ii) RWT photodegradation will be slower than that of fluorescein, making this method useful at multiple time scales.

## Methods

### Study area

We carried out the RWT dye photo-degradation experiment at the Hubbard Brook Experimental Forest (New Hampshire, USA; latitude: 43° 57' 9" N, longitude: 71° 41' 4" W), where the mean annual temperature is 5.6 °C, the mean annual precipitation is 1373 mm, and the mean daily light is 22.1 mol photons m^–2^. We conducted the fluorescein photo-degradation experiment at the Cary Institute of Ecosystem Studies (Millbrook, New York, USA; latitude: 41° 47' 2" N, longitude: 73° 44' 3" W), where the mean annual temperature is 10.1 °C, the mean annual precipitation is 1232 mm, and the mean daily light is 25.5 mol photons m^–2^.

### Ethics statement

No specific permissions were required for these activities. The field studies did not involve endangered or protected species.

### Field experiments

The degradation of RWT and fluorescein dyes depends on the intensity of incident light and on the fraction of incoming light that is absorbed [32]. Based on this relationship, total light irradiance can be related to the photodegradation of the dyes, which can be measured as the decrease in fluorescence. These dyes mainly absorb visible light between 450 and 580 nm (425-525 fluorescein, 475-575 RWT) with some absorption in the UV range [33]. We prepared dye solutions with liquid RWT (Keystone Aniline Corp., Chicago, Illinois, USA) or fluorescein (Pylam Products Company, Tempe, Arizona, USA) diluted with ultrapure water (Millipore Company, Bedford, Massachusetts, USA) to the desired concentration. We used an initial concentration of 75 ppb of both RWT and fluorescein as recommended by Bechtold et al. [16] and did not evaluate effects of concentration on photodegradation. Solutions were kept refrigerated and in the dark before use, then transferred into glass borosilicate scintillation vials. Glass borosilicate scintillation vials permit energy transmission of 90% for PAR (>400 nm), 65% for ultraviolet A radiation (UVAR) (400–320), and <5% for ultraviolet B radiation (UVBR) (<320) [34]. We placed a sub-set of dye solution in glass vials covered with aluminum foil to assess if temperature altered degradation when light was not a factor. We used either three or four replicate vials to assess the effect of light on opaque and clear containers. We monitored the temperature of the dye solution using an additional vial that was either monitored at each sampling period with a digital thermometer (Fisher Scientific Inc.) or every 20 mins by placing a waterproofed iButton temperature logger (iButton DS1921G; Maxim Integrated, San Jose, California, USA) into the solution.

In the summer of 2012, we investigated RWT and fluorescein decay rate across an irradiance gradient from full sunlight to deep shade conditions under tree canopies. Thus, light was naturally filtered by tree canopies, which can absorb or quench red to far-red light wavelengths (610–760 nm) [35] and replicate conditions that are likely to be common in most in field studies.

We measured dye concentrations in subsamples with a fluorescent dye channel (570 nm for RWT and 515 nm for fluorescein) on an AquaFluor Handheld Fluorometer (TurnerDesigns, Sunnyvale, California, USA) at different time intervals. To prevent any disturbance caused by temperature variation, we calibrated the fluorometer before each sampling period with a standard solution that was brought to the same temperature as the samples. Given that RWT photodegrades slower than fluorescein [16], we varied the time of exposure to sunlight for each dye. In this study, RWT was exposed for 23 days and sampled once or twice daily, while fluorescein was exposed to sunlight for 5 hours and sampled every 7-25 minutes.

**Figure 1:**
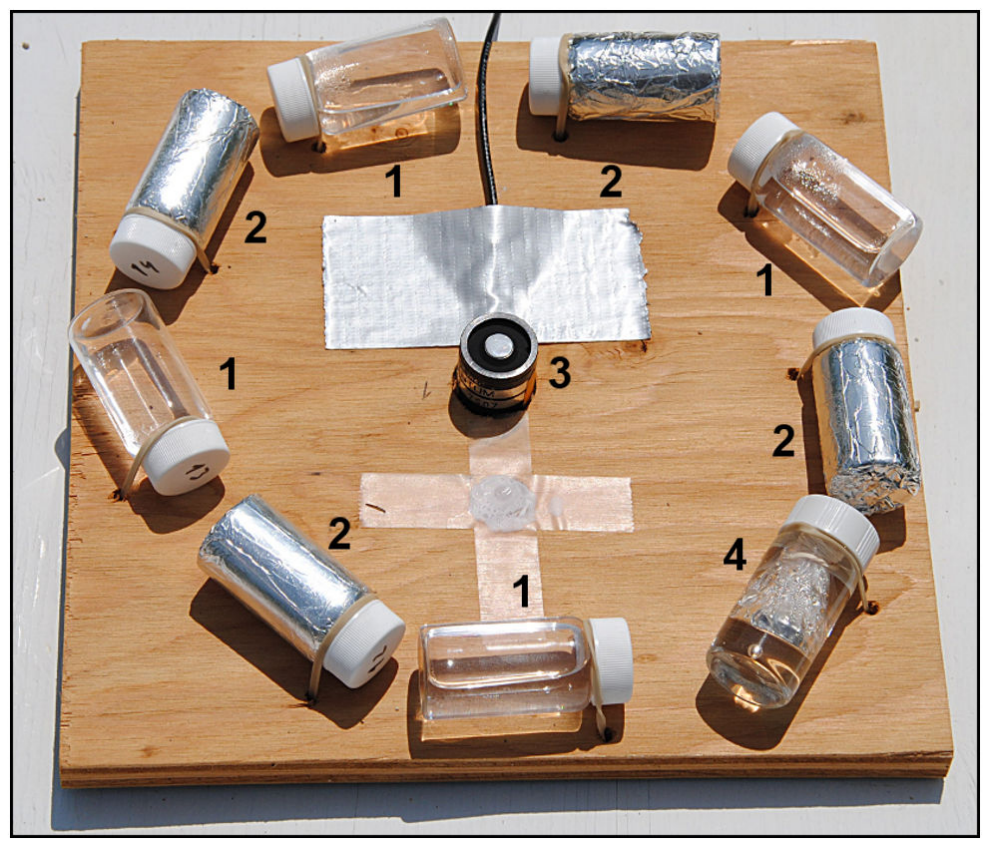
Experimental setup. (1) Set of replicate clear vials containing dye solution, (2) set of replicate opaque vials containing dye solution used as controls, (3) quantum sensor connected to a datalogger, (4) waterproofed iButton temperature logger inside a dye solution vial.

We recorded the amount of accumulated light with PAR quantum sensors (LI-190SA; LI-COR Inc., Lincoln, Nebraska, USA) connected to dataloggers (21x Micrologger; Campbell Scientific Inc., Logan, Utah, USA). Each quantum sensor was surrounded by a set of replicate clear and opaque vials that were attached horizontally to wooden boards 9 cm away from the sensor and placed on level platforms 50 cm above the ground (Figure 1). We programmed dataloggers to average light readings every second for 10 min. This average reading was used as a constant value per second for its corresponding 10-min interval. We then multiplied one-second light values by the corresponding time interval to approximate cumulative irradiance, obtaining units of µmol photons m^–2^.

### Laboratory experiments

Variations in temperature can affect dye fluorescence. Temperature correction coefficients of 2.6% per ºC and 0.36% per ºC have been reported for RWT and fluorescein, respectively [26,27]. Given that our samples were subjected to the higher temperature fluctuations (sun/shade and day/night) that occur on terrestrial surfaces compared to being placed in-stream, we investigated the relationship between RWT and fluorescein concentrations and temperature by placing 10 replicate samples of each dye type into growth chambers (Percival Incubators, Perry IA, USA). We recorded dye temperature every 3 minutes using an iButton logger placed inside a vial. Initial dye concentration was recorded at 20 °C and samples were then placed immediately into dark growth chambers and heated to 45 °C for 5 hours. We removed samples and recorded dye fluorescence at 45 °C and then allowed samples to cool to 20 °C and recorded the final fluorescence at this temperature.

### Statistical analysis

We related the decay coefficient (k) to accumulated light instead of time given that dye degradation is a light dependent process. From the regression of dye concentration versus accumulated light we obtained that the best fit for *k* followed an exponential decay curve based on 1^st^-order kinetics, confirming findings from other authors [36]. The resulting *k* would be:

$${\left[dye\right]}_{L}={\left[dye\right]}_{initial}e^{-k\left(light\right)}$$

where [dye]_*L*_ = concentration of dye (µg/L) at a measured light intensity, [dye]_*initial*_ = initial concentration of dye (µg/L), *light* = measured light intensity (µmol photons m^–2^), *k* = photodecay coefficient of that dye/µmol photons m^–2^.

We also calculated half-life of the dyes in terms of accumulated light (amount of light required for the dye to degrade to one half of its initial value, *l*_1/2_). All analyses were carried out using SPSS (version 15; IBM SPSS Statistics) with α = 0.05.

## Results

**Figure 2:**
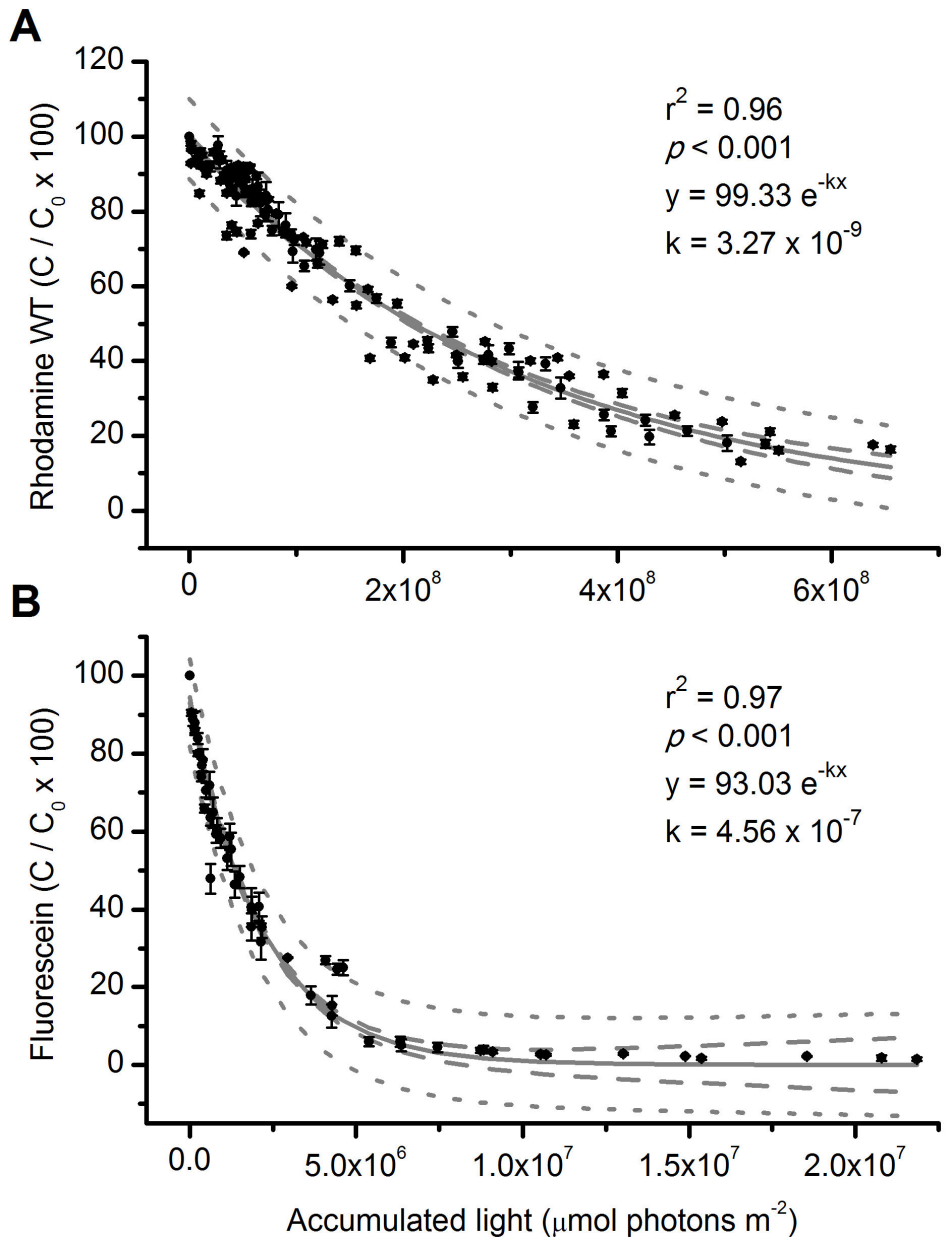
Rhodamine WT and fluorescein photodegradation after exposure to solar radiation. Mean concentration (C/C_0_ x 100) of rhodamine WT (A) and fluorescein (B) solutions in glass vials exposed to solar radiation over 17 d (rhodamine WT) or 5 h (fluorescein). Error bars are 1 SE. Dashed lines indicate 95% confidence intervals and dotted lines indicate prediction intervals.

**Figure 3:**
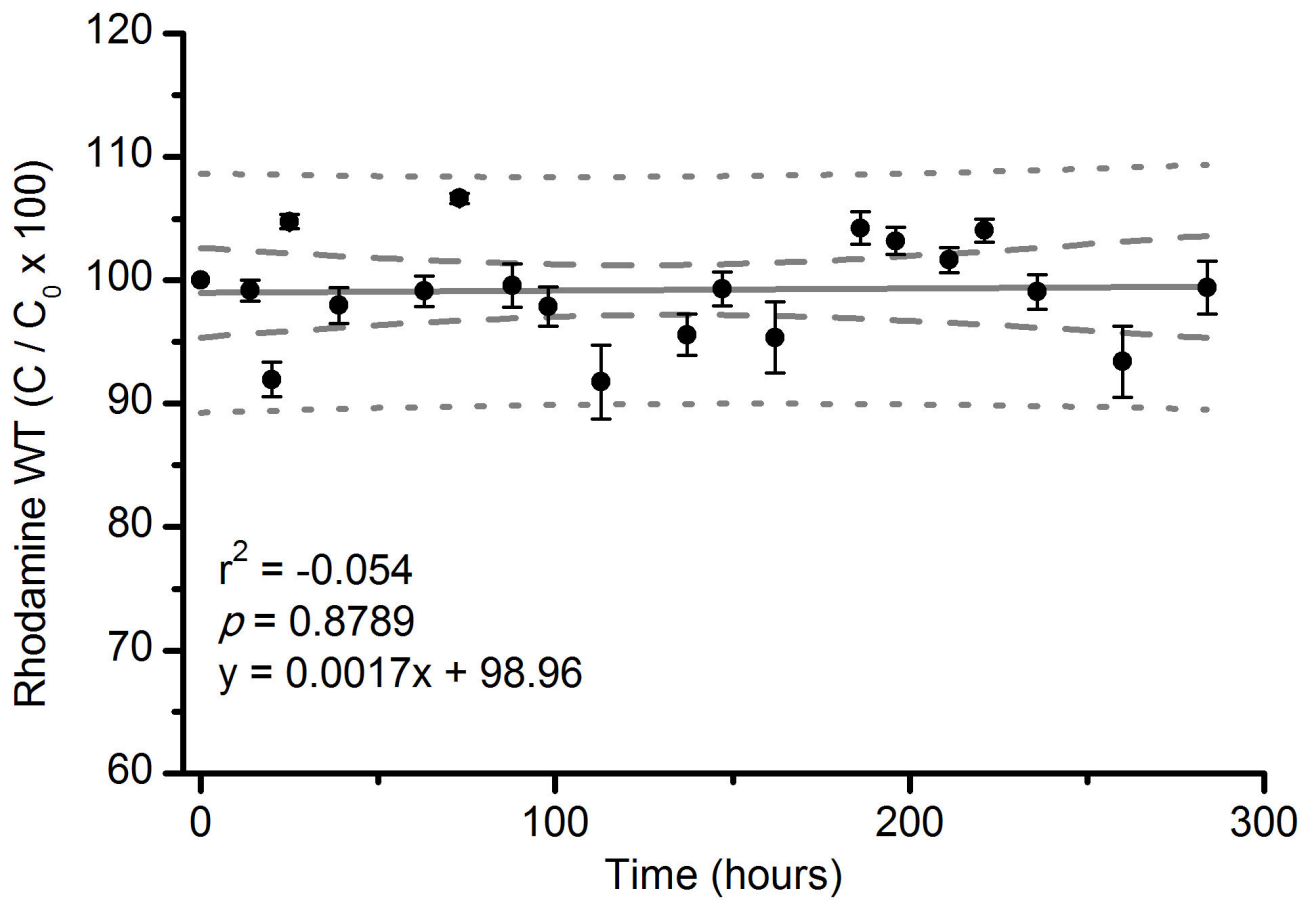
Effect of natural temperature fluctuations on the degradation of RWT. Mean concentration (C/C_0_ x 100) of rhodamine WT solutions in opaque glass vials exposed to solar radiation over 12 d. Error bars are 1 SE. Dashed lines indicate 95% confidence intervals and dotted lines indicate prediction intervals.

Rhodamine WT and fluorescein photodegraded after exposure to both direct sunlight and shaded conditions below forest canopies. We found a significant negative exponential relationship between average dye concentration and accumulated light. Decay constants for both shaded and unshaded dye vials were 3.27 x 10^-9^ for RWT (R^2^ = 0.96, DF = 2, F = 11989.85, *p* < 0.001; Figure 2A), and 4.56 x 10^-7^ (R^2^ = 0.97, DF = 2, F = 2953.02, *p* < 0.001; Figure 2B) for fluorescein. The half-life of RWT was longer (2.08 x 10^8^ µmol photons m^-2^) than fluorescein (1.35 x 10^6^ µmol photons m^-2^) after exposure to sunlight. The concentration of RWT placed in opaque vials (protected from sunlight) did not change after 12 days of exposure to natural sunlight and temperature fluctuations (Figure 3).

**Figure 4:**
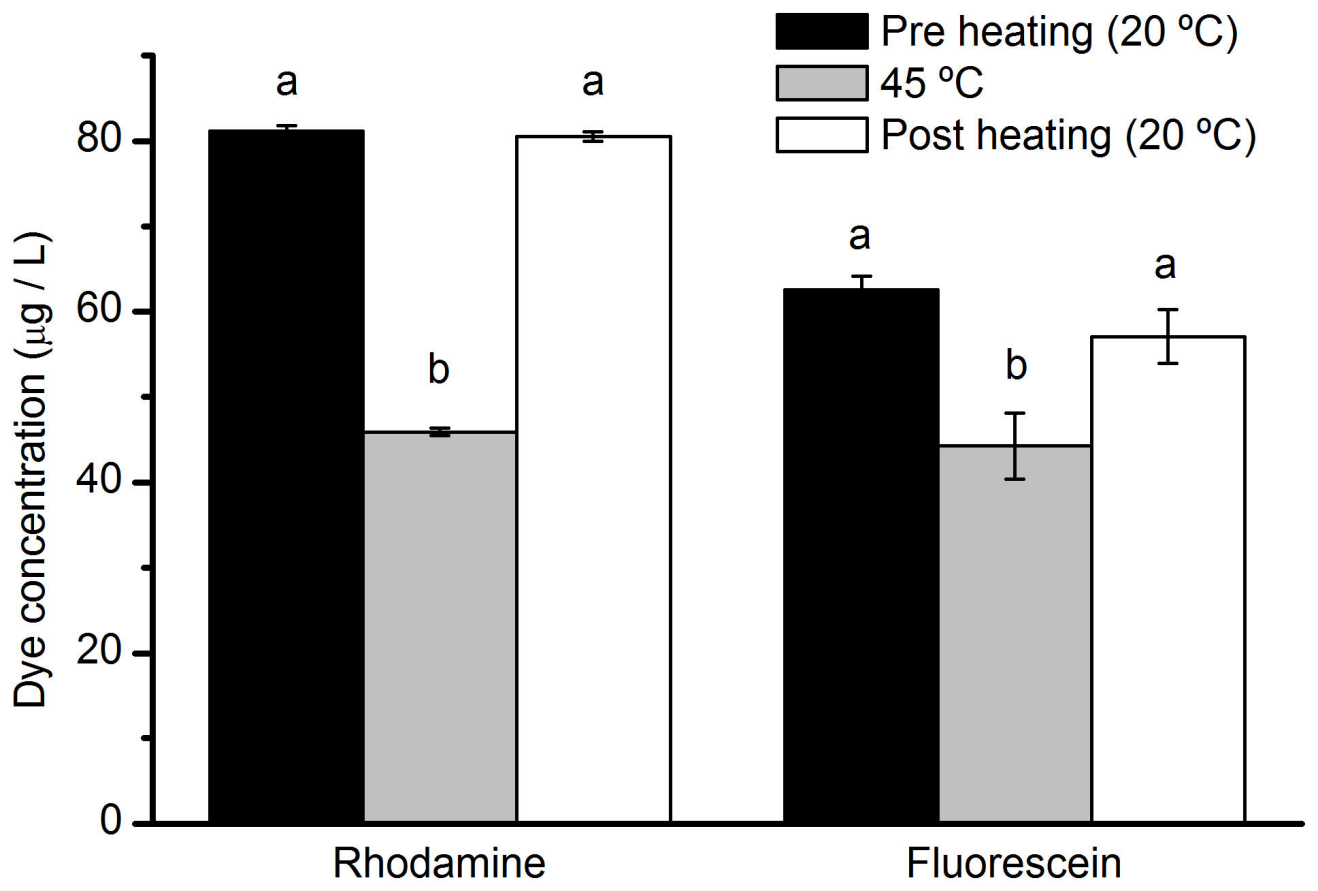
Temperature effect on fluorescence and degradation of RWT and fluorescein. Mean concentration (µg / L) of rhodamine WT and fluorescein solutions in glass vials in the dark before (20 °C), during (45 °C) and after heating (20 °C). Different letters among bars indicate significant differences among treatments for each dye. Total time of heating = 5 h. Error bars are 1 SE.

Fluorescence of both dyes decreased when temperatures increased but photodegradation was not altered. Dye fluorescence was significantly higher at 20 °C than at 45 °C for both RWT (DF = 2, F = 1310.81, p < 0.001; Figure 4), and fluorescein (DF = 3, F = 7.867, p < 0.001; Figure 4). However, fluorescence at 20 °C after a heating treatment (5 hours at 45 °C) was not significantly different from pre-heating fluorescence at 20 °C for either dye (Figure 4).

## Discussion

Rhodamine WT and fluorescein dyes degraded upon exposure to solar radiation but at different rates. Samples exposed to the same amount of accumulated light over different periods of time (sun *vs.* shade conditions) displayed the same rates of decomposition, indicating that the degradation of dye molecules was independent of time. This suggests that both dyes can be used to measure light in places that have varying light regimes [16], such as grasslands (high light) and forests (low light). Furthermore, our results show that dyes are stable in varied light regimes and when they are exposed to different temperatures, which suggests that differences in the number of light hours, cloud coverage, or temperature minimally affect this method.

Fluorescein degrades faster than RWT under similar conditions [19]. In our experiments, RWT degraded 85% after 17 days of exposure, while fluorescein experienced the same degradation after 1 hour. The time period that RWT was exposed to light included nights and cloudy days with less irradiance, so the inclusion of total or partially dark hours in degradation time may underestimate speeds of RWT degradation. To compare degradation rates between the two dyes, we calculated *l*_1/2_ (see Methods section). The *l*_1/2_ of RWT was 157 times longer than that of fluorescein in terms of decay with the amount of accumulated light. This suggests that RWT may be the best choice for light measurement during long-term experiments (i.e., weeks to months) where there is a need to integrate variability caused by cloud coverage or dense forest canopies that create spatially variable light conditions [10,12]. Under high light conditions that accelerate the degradation process, RWT would also be the preferred dye. Fluorescein may be more useful under low irradiance conditions where RWT would take too long to degrade significantly, or for short-term experiments (hrs to days). A combination of both dyes would give complementary information and provide a comprehensive analysis of incoming light.

The concentration of RWT in opaque vials did not change after 12 days of exposure to temperature fluctuations. This lack of degradation suggests that degradation of this dye is not altered by temperature. The fluorescence of both RWT and fluorescein decreased when heated, and confirm that fluorescence is affected by temperature [26,27] and can be corrected using correction curves [26,37]. However, our additional temperature study indicates that cooling dye back to the initial temperature results in no change in fluorescence and further indicates that there was no degradation of dye molecules in our temperature range, making the correction curves unnecessary. Thus, it is an important part of this method to account for temperature by measuring samples at the same temperature, and calibrating the fluorometer with a standard at the same temperature of the samples.

The equations that correlate dye degradation with accumulated light may be used by researchers intending to use this method without any adjustments provided that the experimental setup is similar to the one presented in this work. In case of experimental variations, the equations should be adjusted to account for the new conditions. Parameters such as dye concentration or temperature are critical, but other factors such as the container used for the dyes during the experiments (different transmission rates of light depending on the material) or the fluorometer (or its calibration) may also influence the results. A reduced version of our experiments with only a few samples would be enough to adjust the equations that correlate dye degradation with accumulated light to different experimental conditions. Also, using the dye degradation as a relative measurement of incoming solar radiation can be a simpler alternative when the calculation of accumulated light is not required.

One of the advantages of this method, compared to those based on PAR sensors with their corresponding data loggers, is its low cost. Given the diversity and variability in the price of scientific equipment, it is difficult to give an approximation of the price of each system. We estimate that for one single measurement the average cost of our method, including dyes, the fluorometer and other accessories, would be one half of that based on PAR sensors. However, the main advantage of our method is the ability to sample several areas simultaneously. For example, creating a light map based on 100 simultaneous measurements would require 100 PAR sensors which would likely be prohibitively expensive. With photodegrading dyes, the cost would be minimum, at least 200 times cheaper than using conventional light meters.

We conclude that RWT and fluorescein can be used to estimate light under a varying range of light conditions in terrestrial ecosystems. Both dyes are economical and the data obtained from measurements is easy to interpret. This method is particularly useful to integrate solar radiation over time and to measure light simultaneously at different locations, and might be a better alternative to expensive and time consuming traditional light measurement methods. The accuracy, low price and ease of the method proposed here make it a powerful tool for extensive sampling of large areas. Improved characterization of light regimes in terrestrial ecosystems and development of high resolution light maps are possible with this method.
